# c-MYC and reactive oxygen species play roles in tetrandrine-induced leukemia differentiation

**DOI:** 10.1038/s41419-018-0498-9

**Published:** 2018-04-27

**Authors:** Guixian Wu, Ting Liu, Han Li, Yafang Li, Dengju Li, Wenhua Li

**Affiliations:** 1 Hubei Key Laboratory of Cell Homeostasis, College of Life Sciences, Wuhan University, Wuhan, 430072 P. R. China; 2 Department of Hematology, Tongji Hospital of Tongji Medical College, Huazhong University of Science and Technology, Wuhan, P. R. China

## Abstract

Tetrandrine is a broadly used bisbenzylisoquinoline alkaloid component of traditional Chinese medicine that has antitumor effects in some cancer types. In this study, we investigated the effects of tetrandrine on leukemia in vitro and in vivo. The results showed that tetrandrine effectively induced differentiation and autophagy in leukemia cells. In addition, tetrandrine treatment activated the accumulation of reactive oxygen species (ROS) and inhibited c-MYC protein expression. Further, we found that treatment with the ROS scavengers N-acetyl-L-cysteine (NAC) and Tiron as well as overexpression of c-MYC reduced tetrandrine-induced autophagy and differentiation. Moreover, a small molecular c-MYC inhibitor, 10058-F4, enhanced the tetrandrine-induced differentiation of leukemia cells. These results suggest that ROS generation and c-MYC suppression play important roles in tetrandrine-induced autophagy and differentiation, and the results from in vivo experiments were consistent with those from in vitro studies. Therefore, our data suggest that tetrandrine may be a promising agent for the treatment of leukemia.

## Introduction

Leukemia is a disease caused by malignant proliferation of hematopoietic stem cells. The most important characteristic of leukemia is that cells are blocked at an early stage of development and fail to differentiate into functional mature cells^1^. In the 1970s and 1980s, studies showing the capabilities of certain chemicals to induce the differentiation of leukemia cell lines fostered the concept of treating leukemia by forcing malignant cells to undergo terminal differentiation instead of killing them through cytotoxicity^2,3^. The best proof of principle for differentiation therapy has been the treatment of acute promyelocytic leukemia (APL) with all-trans retinoic acid (ATRA)^4–7^. Although various chemicals are used to treat leukemia, tumor resistance and the cytotoxicity of many drugs have prompted the search for new therapeutic agents.

Tetrandrine is a bisbenzylisoquinoline alkaloid isolated from the roots of the traditional Chinese medicine plant Stephaniae tetrandrae. Tetrandrine has been broadly used for anti-allergic, anti-inflammatory and anti-silicosis treatments^2,8,9^. Some studies have shown that tetrandrine can inhibit proliferation and induce apoptosis in lung carcinoma, bladder cancer and colon cancer^10–12^. We have reported that relatively high concentrations of tetrandrine induce apoptosis through the reactive oxygen species (ROS)/Akt pathway and that low doses of tetrandrine trigger autophagy via ATG7 and the ROS/ERK pathway in human hepatocellular carcinoma^13,14^. These studies suggest that tetrandrine can exhibit strong antitumor effects and has potential as a cancer chemotherapeutic agent.

Autophagy, which is a dynamic process induced by starvation or cellular stress, is essential for cell differentiation, cell survival, aging and the cell cycle^15–17^. Although autophagy is a self-protecting mechanism regulated by nutritional deficiencies, excessive autophagy leads to cell death^18^. In recent years, autophagy was found to be closely related to cancer^19^, and ATG7 or ATG4B knockdown has been reported to alter the viability of primary chronic myeloid leukemia CD34+ progenitor cells. Many studies have shown that autophagy is important for myeloid cell differentiation^20–24^. Hence, enhanced autophagy may be a promising treatment to promote differentiation in leukemia patients.

In our study, we investigated the mechanism of tetrandrine-induced leukemia differentiation in vitro and in vivo. Our results demonstrated that tetrandrine triggered autophagy, induced ROS generation, and inhibited c-MYC expression, which can regulate differentiation. These findings suggest that tetrandrine may be a promising agent for leukemia treatment.

## Results

### Tetrandrine inhibited cell proliferation in leukemia cells

**Fig. 1:**
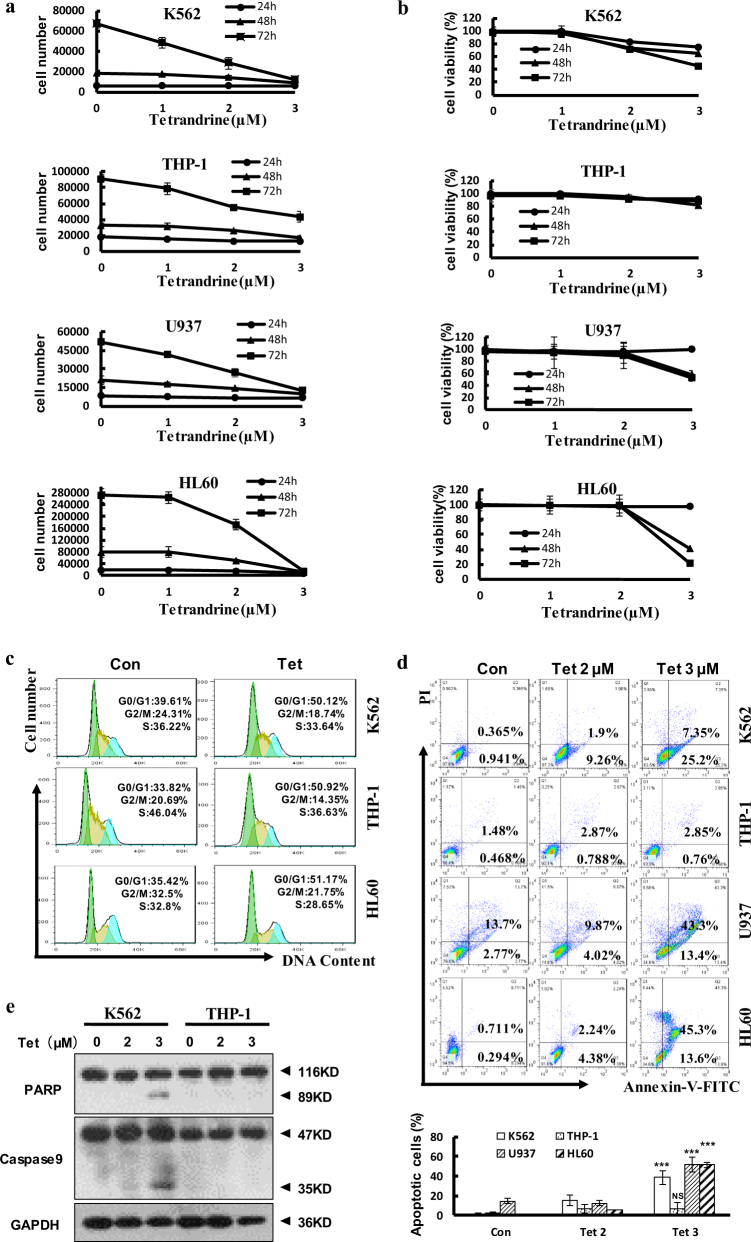
Tetrandrine at 2 µM inhibited leukemia cell proliferation but did not induce apoptosis. DMSO was used as a negative control (Con). The data are presented as the mean ± S.D. (**a**) Cells were treated with tetrandrine (0, 1, 2 or 3 μM) for 24 h, 48 h and 72 h and then cell proliferation was assessed using a cell counting method. (**b**) Cell viability was determined by the trypan blue dye-exclusion assay. *n* = 3. (**c**) After 48 h of treatment with DMSO or 2 μM tetrandrine, cells were stained with PI and the cell cycle stage was determined by flow cytometry. (**d**) The cells were treated with tetrandrine (0, 2, or 3 μM) for 48 h. Apoptotic cells were detected by flow cytometry. *n* = 3. ***p < 0.001, NS, not significant. (**e**) Western blot analysis of the PARP and caspase-9 expression in the cells after tetrandrine (0, 2 or 3 μM) treatment for 48 h. GAPDH was used as a loading control

First, leukemia cells were counted to examine the effects of tetrandrine on leukemia cell proliferation, and the results suggested that 2 μM and 3 μM tetrandrine dramatically inhibited cell proliferation (Fig. 1a). However, cell viability analysis demonstrated that 0–2 μM tetrandrine did not increase cell death (Fig. 1b). To further investigate proliferation inhibition, cell cycle analysis was performed and showed significant cell cycle arrest at G0/G1 phase (Fig. 1c), the statistic analysis was shown in Figure S1. Moreover, cell apoptosis analysis by flow cytometry indicated that 2 μM tetrandrine did not kill cells (Fig. 1d), and western blot analysis of PARP and caspase-9 expression revealed similar results (Fig. 1e). In conclusion, 2 μM tetrandrine inhibited proliferation but did not induce apoptosis in leukemia cells.

### Tetrandrine treatment induced differentiation in leukemia cells

**Fig. 2:**
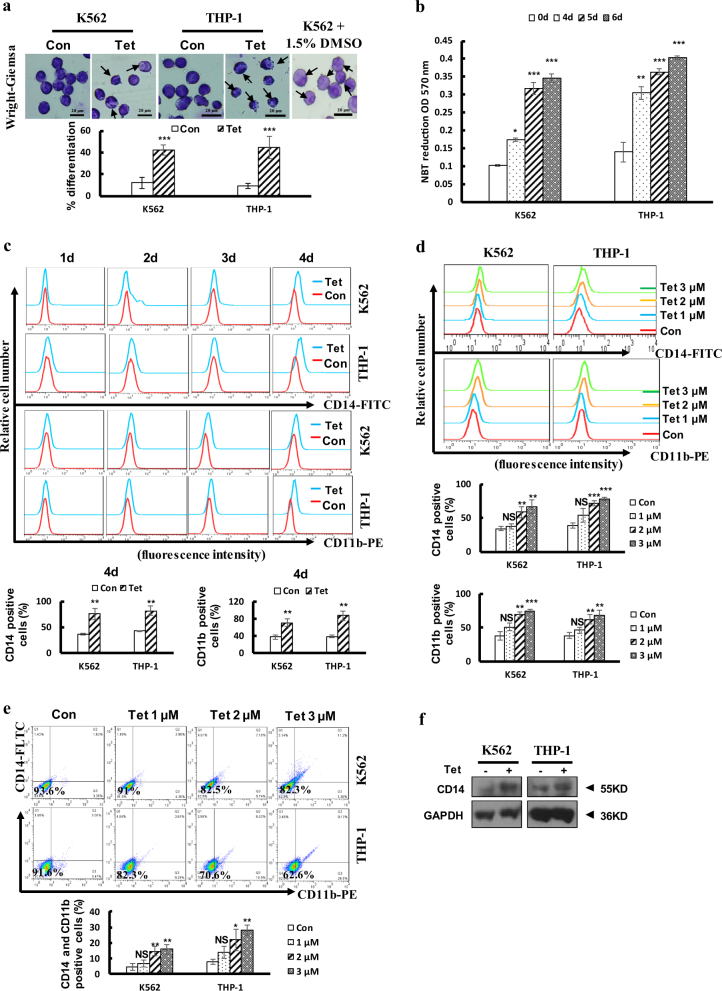
Tetrandrine at 2 μM induced differentiation in leukemia cells. The bars indicate the S.D. *p < 0.05, **p < 0.01, ***p < 0.001, NS, not significant. (**a**) The morphology of the Wright-Giemsa-stained cells was observed under a microscope after the cells were treated with DMSO or 2 μM tetrandrine for 4 days. The 1.5% DMSO-treated K562 cells was used as positive control. The arrows indicate lower nucleus/cytoplasm ratio and the cells with horseshoe shape nuclei. *n* = 3. (**b**) NBT reduction assay detected cell differentiation after the cells were treated with 2 μM tetrandrine for 0, 4, 5 and 6 days. (**c**) CD14 or CD11b antigen expression were measured after the cells were treated with DMSO or 2 μM tetrandrine treatment at the indicated times (**d**) or were measured by flow cytometry after tetrandrine treatment at the indicated concentrations for 4 days. (**e**) CD14 and CD11b expression was co-detected by flow cytometry after 4 days of 0, 1, 2 or 3 μM tetrandrine treatment. (**f**) CD14 and GAPDH levels were measured by western blot after the cells were treated with DMSO or 2 μM tetrandrine for 4 days

Differentiation therapy can improve the cure rates of patients with leukemia^1^. To investigate whether tetrandrine induces differentiation in leukemia cells, we used Wright-Giemsa staining to detect the morphologic features of tetrandrine-treated cells. The 1.5% DMSO-treated K562 cells was used as a positive control^25^. The results revealed that tetrandrine-treated cells showed a highly differentiated cellular morphology, such as a lower nucleus/cytoplasm ratio and the cells with horseshoe shape nuclei (Fig. 2a). Then, we found that tetrandrine-treated cells exhibited increased nitroblue tetrazolium (NBT) reduction in a time-dependent manner (Fig. 2b). Moreover, flow cytometry showed that tetrandrine remarkably increased CD14 and CD11b expression levels in a time- and dose-dependent manner (Fig. 2c, d). Next, flow cytometry analysis showed that tetrandrine-treated cells expressed higher levels of CD14 and CD11b than DMSO-treated cells when cells were co-stained with CD14 and CD11b (Fig. 2e). Finally, western blot analysis also showed that CD14 expression was remarkably higher in tetrandrine-treated cells than in DMSO-treated control cells (Fig. 2f). Altogether, these findings strongly suggest that tetrandrine induced leukemia differentiation.

### Tetrandrine-facilitated differentiation was related to autophagy

**Fig. 3:**
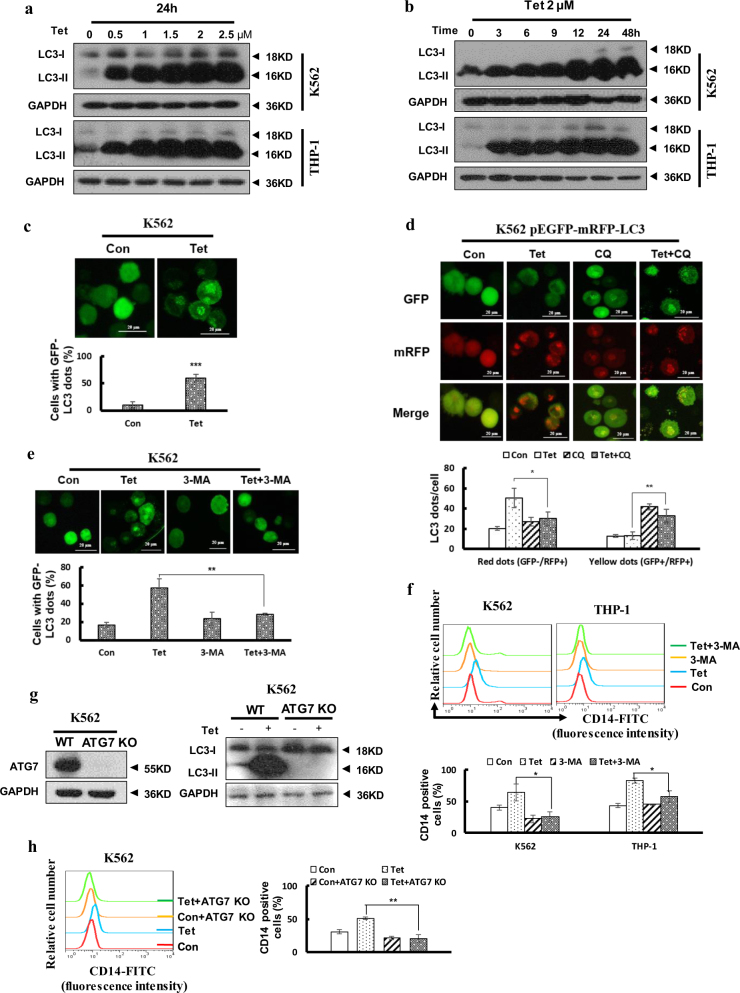
Autophagy played an important role in tetrandrine-induced differentiation. The bars indicate the S.D. (**a**) K562 and THP-1 cells were treated with the indicated concentration of tetrandrine for 24 h or (**b**) with 2 μM tetrandrine for the indicated durations. Western blot analysis of LC3 levels. GAPDH was used as a loading control. (**c**) K562 cells were transfected with the GFP-LC3 plasmid for 24 h, subsequently treated with DMSO or 2 μM tetrandrine for 24 h and observed by fluorescence microscopy. Representative experiments are shown to indicate the cellular localization patterns of the GFP-LC3 fusion protein (magnification ×400), and percentage of cells with GFP-LC3 puncta were used to quantify the percentage of autophagic cells. *n* = 3, ***p < 0.001. (**d**) After a 1 h pretreatment with 10 μM CQ and 48 h of subsequent treatment with DMSO or 2 μM tetrandrine, fluorescence microscopy detected green and red fluorescent spots in K562 cells transfected with the ptfLC3 plasmid and percentage of cells with red dots were used to quantify the autophagic flux, while yellow dots (the overlay of green and red fluorescence) were increased if the process of autophagosomes fusing with lysosomes was inhibited. *n* = 3, *p < 0.05, **p < 0.01. (**e**) GFP-LC3-transfected cells were pretreated with or without 1.5 mM 3-MA for 1 h. The cells were then exposed to DMSO or 2 μM tetrandrine for 24 h, and the localization of GFP-LC3 was observed using a fluorescent microscope (magnification ×400). *n* = 3, **p < 0.01. (**f**) CD14 antigen expression was analyzed by flow cytometry after treatment for 4 days with or without pretreatment with 1.5 mM 3-MA for 1 h, and a statistic analysis for three experiments in the bottom. *p < 0.05. (**g**) The expression of ATG7 and LC3-II proteins in K562 ATG7 KO cells were assessed via western blot, and K562 WT cells were used as a positive control. (**h**) CD14 expression was determined by flow cytometry in K562 ATG7 and K562 WT cells after treatment for 4 days, and a statistic analysis for three experiments in the right. **p < 0.01. WT wild type

In recent years, several agents that induce cell differentiation and cell death have been shown to activate autophagy^26,27^. We examined whether tetrandrine could induce autophagy in leukemia cells. Western blot analysis showed that tetrandrine remarkably increased LC3-II protein levels in a dose- and time-dependent manner (Fig. 3a, b). And acridine orange staining assay demonstrated that the intensity of acridine orange red staining was significantly enhanced in the tetrandrine-treated cells (Figure S2A and B). To confirm tetrandrine-induced autophagy, K562 cells were transfected with the GFP-LC3 plasmid and treated with tetrandrine, followed by visualization of the punctate fluorescent pattern of GFP-LC3 by fluorescence microscopy (Fig. 3c). Further, tetrandrine-induced autophagy flux was investigated by expression ptfLC3 plasmid and with or without CQ. After 48 h treatment, tetrandrine-treated cells showed only mRFP-LC3 dots, and tetrandrine combine CQ-treated cells showed both mRFP and GFP-LC3 dots, which indicated that tetrandrine induced autophagy flux been blocked by CQ (Fig. 3d). To examine the relationship between autophagy and differentiation in tetrandrine-induced cells, 3-methyladenine (3-MA) was used to inhibit autophagy, revealing that the tetrandrine-mediated autophagy process and CD14 expression were strongly prevented (Fig. 3e, S2C-E and 3F). Next, we also found that tetrandrine-treated cell differentiated morphology features were restored by 3-MA (Figure S2F). To further determine autophagy was involved in tetrandrine-induced differentiation, ATG7 knockout K562 cells were used (Fig. 3g). Flow cytometry detection showed that tetrandrine-induced CD14 expression was effectively inhibited in ATG7 knockout K562 cells (Fig. 3h). These data suggest that tetrandrine induces autophagy of leukemia cells and that autophagy plays a critical role in the tetrandrine-induced differentiation.

### Tetrandrine promoted intracellular ROS accumulation, an important event in tetrandrine-induced autophagy and differentiation

**Fig. 4:**
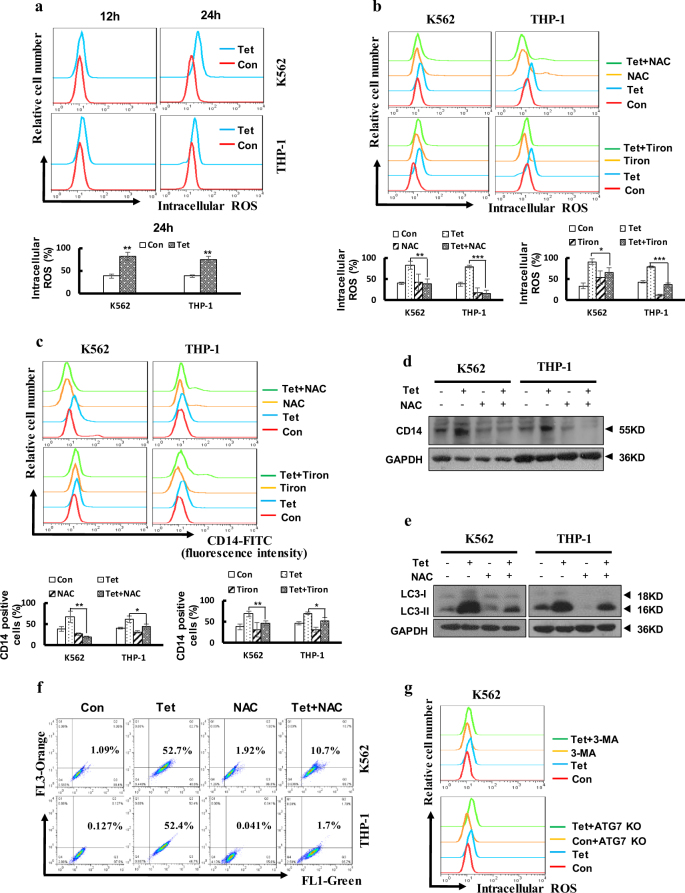
Tetrandrine induced accumulation of intracellular ROS, leading to cell autophagy and differentiation. The bars indicate the S.D. *p < 0.05, **p < 0.01, ***p < 0.001. (**a**) Intracellular ROS levels were assessed by flow cytometry after treatment with DMSO or 2 μM tetrandrine for 12 h or 24 h. (**b**) The cells were pretreated with NAC (K562 with 15 mM and THP-1 with 10 mM) and Tiron (K562 with 0.5 mM and THP-1 with 0.2 mM) for 1 h, and then treated with DMSO or 2 μM tetrandrine for 24 h. The intracellular ROS levels were measured by flow cytometry (**c**) or CD14 expression was measured by flow cytometry after 4 days of treatment. (**d**) CD14 and GAPDH levels were analysis by western blot after the indicated treatments for 4 days. (**e)** After 24 h of treatment, the LC3 and GAPDH levels were measured by western blots and (**f**) cells were stained with acridine orange and then analyzed by flow cytometry. (**g**) After 24 h of DMSO or 2 μM tetrandrine treatment, flow cytometry was used to detect the generation of ROS in K562 ATG7 KO cells and K562 cells pretreated with 1.5 mM 3-MA

Some reports have suggested that ROS may be involved in cell differentiation^28^. Therefore, we aimed to determine whether tetrandrine-induced differentiation was associated with intracellular ROS activation. As shown in Fig. 4a, we found that tetrandrine-treated cells had higher intracellular ROS levels than DMSO-treated cells. To further investigate whether ROS accumulation was involved in tetrandrine-induced differentiation, CD14 expression was assessed after tetrandrine treatment in the presence or absence of N-acetyl-L-cysteine (NAC) and Tiron. The results indicated that tetrandrine-induced ROS generation and differentiation were markedly restrained by NAC and Tiron (Fig. 4b, c), and western blot analysis of CD14 expression revealed similar results (Fig. 4d). Recent studies have shown that ROS can initiate autophagosome formation. Therefore, LC3-II levels and acridine orange-positive cells were examined with or without the addition of NAC. Notably, the results shown that NAC remarkably inhibited the increased LC3-II levels and the intensity of acridine orange red staining in the tetrandrine-treated cells (Fig. 4e, f and S3). Importantly, ROS accumulation was detected in the cells in which autophagy was inhibited (Fig. 4g). Thus, these data suggest that ROS generation may mediate autophagy and differentiation in response to tetrandrine-treated leukemia cells.

### Inhibition of c-MYC expression facilitated tetrandrine-induced differentiation

**Fig. 5:**
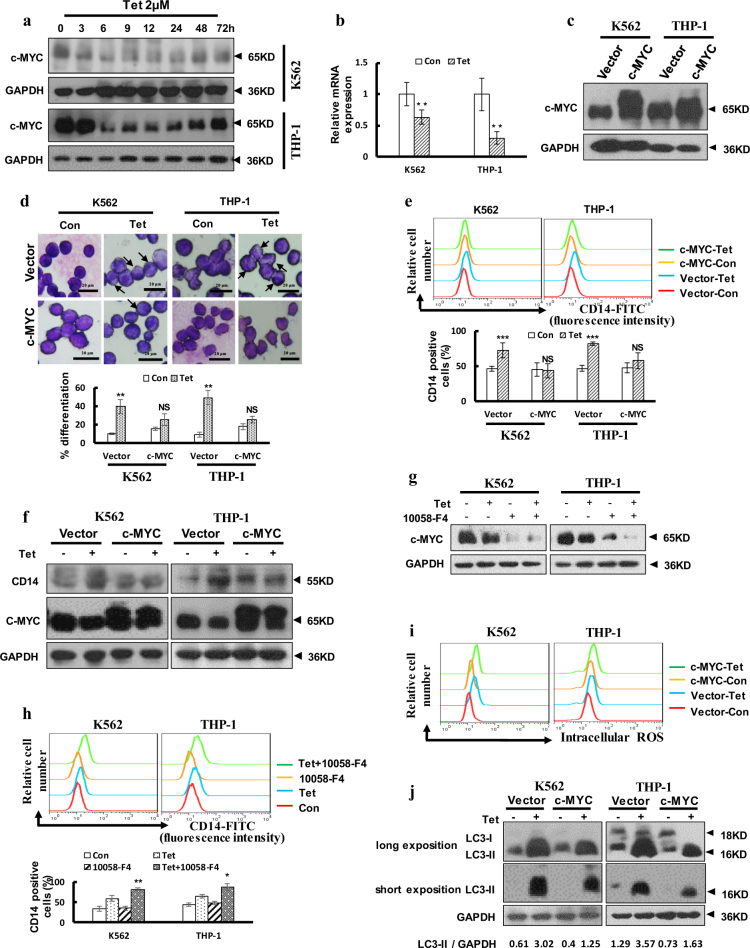
Tetrandrine induced differentiation by inhibiting c-MYC expression. The bars indicate the S.D. (**a**) Western blot analysis of c-MYC and GAPDH levels in cells treated with 2 μM tetrandrine for different durations. (**b**) qRT–PCR analysis of c-MYC expression in cells after 24 h of treatment. *n* = 3. **p < 0.01. (**c**) Western blot analysis of c-MYC and GAPDH levels in cells overexpressing either vector or c-MYC. (**d**) Wright-Giemsa staining was used to assess cell morphology after 4 days of DMSO or 2 μM tetrandrine treatment in cells overexpressing either vector or c-MYC. The arrows indicate lower nucleus/cytoplasm ratio and the cells with horseshoe shape nuclei. **p < 0.01, NS, not significant. (**e**) After 4 days treatment, CD14 expression was detected by flow cytometry and (**f**) the CD14 and c-MYC levels were measured by western blot. *n* = 3. ***p < 0.001, NS, not significant. (**g**) The cells were pretreated with 40 μM 10058-F4 and then treated with DMSO or 2 μM tetrandrine for 24 h. Western blot analysis of c-MYC and GAPDH levels. (**h**) Flow cytometry was used to analyze CD14 expression after the indicated treatments for 4 days. (**i**) Intracellular ROS was assessed via FACS analysis in cells overexpressing either vector or c-MYC after treatment with DMSO or 2 μM tetrandrine for 24 h. (**j**) Western blot analysis of LC3 and GAPDH levels

Recently, deregulated or elevated expression of c-MYC has been reported in many cancers, and sustained activation of c-MYC can inhibit terminal differentiation^29^. In our study, we treated cells with tetrandrine for various treatment durations. Western blot analysis indicated that tetrandrine dramatically suppressed c-MYC expression (Fig. 5a). Moreover, tetrandrine also decreased c-MYC mRNA expression (Fig. 5b). To further establish the role of c-MYC in tetrandrine-induced differentiation, c-MYC was stably expressed in K562 and THP-1 cells (Fig. 5c). As shown in Fig. 5d, the tetrandrine-induced cell morphology features were restored by overexpression of c-MYC. Flow cytometry analysis showed that tetrandrine-induced CD14 expression was prevented in cells overexpressing c-MYC (Fig. 5e), and western blot analysis for CD14 revealed similar results (Fig. 5f). To examine whether reduction of c-MYC activity can enhance tetradrine-induced differentiation, c-MYC inhibitor 10058-F4 was used to co-treatment with tetrandrine. As shown in Fig. 5g, h, though 40 μM 10058-F4 can’t induce cell differentiation, tetrandrine treatment combined with 40 μM 10058-F4 can inhibit c-MYC expression and promote CD14 expression, revealing that further inhibition of c-MYC expression can promote cell differentiation. However, tetrandrine-induced ROS accumulation was still activated in the overexpression c-MYC cells (Fig. 5i). Further, western blot analysis indicated that c-MYC expression inhibited the LC3-II accumulation induced by tetrandrine (Fig. 5j). Detection of tetrandrine-induced acridine orange-positive cells further confirmed that expression of c-MYC may be associated with autophagy (Figure S4). The above results suggest that tetrandrine-induced differentiation was associated with c-MYC expression.

### Tetrandrine induced differentiation in an in vivo xenograft model

**Fig. 6:**
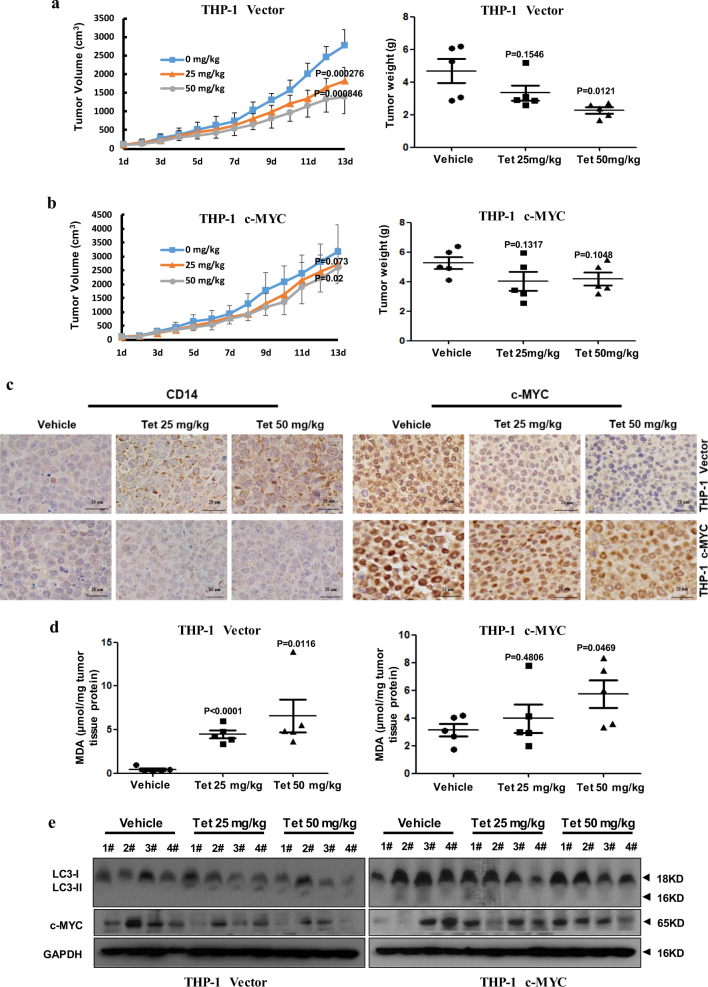
Tetrandrine induced differentiation in an in vivo xenograft model. The bars represent the mean ± S.D. (**a**), (**b**) Tumor volume was measured daily. The tumors were removed by dissection and weighed after 13 days of treatment. (**c**) CD14 and c-MYC expression were evaluated by immunohistochemistry analysis in tumor tissues. Magnification: ×400. (**d**) Tumor tissue proteins were extracted from the THP-1 vector and c-MYC xenografts and were subjected to MDA assay to analyze tissue ROS levels. (**e**) Western blot analysis of c-MYC, LC3 and GAPDH levels in tumor tissues

To investigate the effects of tetrandrine on differentiation in vivo, we established a subcutaneous tumor xenograft model in athymic nude mice using the THP-1 cells overexpressing c-MYC or a control vector. A week later, mice were intragastrically administered vehicle or tetrandrine (25 or 50 mg/kg body weight) for 13 days, and their body weight and tumor size were measured daily. Importantly, tetrandrine treatment reduced tumor growth, as evidenced by the reduction in the tumor volume and lower tumor weight in mice treated with the THP-1 cells overexpressing the control vector compared to those in the mice treated with vehicle, but had a weak effect on tumors wherein c-MYC was overexpressed (Fig. 6a, b). Notably, we found that tetrandrine treatment was well tolerated by all mice as the animals did not display weight loss (Figure S5A). Immunohistochemistry results showed that tetrandrine effectively promoted CD14 expression and decreased c-MYC expression in the mice with THP-1 cells overexpressing the control vector but had minimal effects on tumors overexpressing c-MYC (Fig. 6c). Moreover, the levels of the lipid peroxidation product malondialdehyde (MDA), which was used as a presumptive measure of the ROS levels, were higher in both the THP-1 vector and THP-1 c-MYC tumor tissues following tetrandrine treatment than in those treated with vehicle (Fig. 6d). Finally, decreased c-MYC and increased LC3-II protein levels were detected by western blot in the tetrandrine-treated THP-1 vector tumors (Fig. 6e). Immunohistochemistry also showed more LC3 protein in the tetrandrine-treated THP-1 vector tumors than THP-1 c-MYC tumors (Figure S5B). These results demonstrate that tetrandrine exhibited good antitumor activity in vivo, and the potential mechanism was associated with the induction of tumor cells autophagy and differentiation by inhibiting c-MYC expression.

### Tetrandrine affected differentiation in AML patient cells

**Fig. 7:**
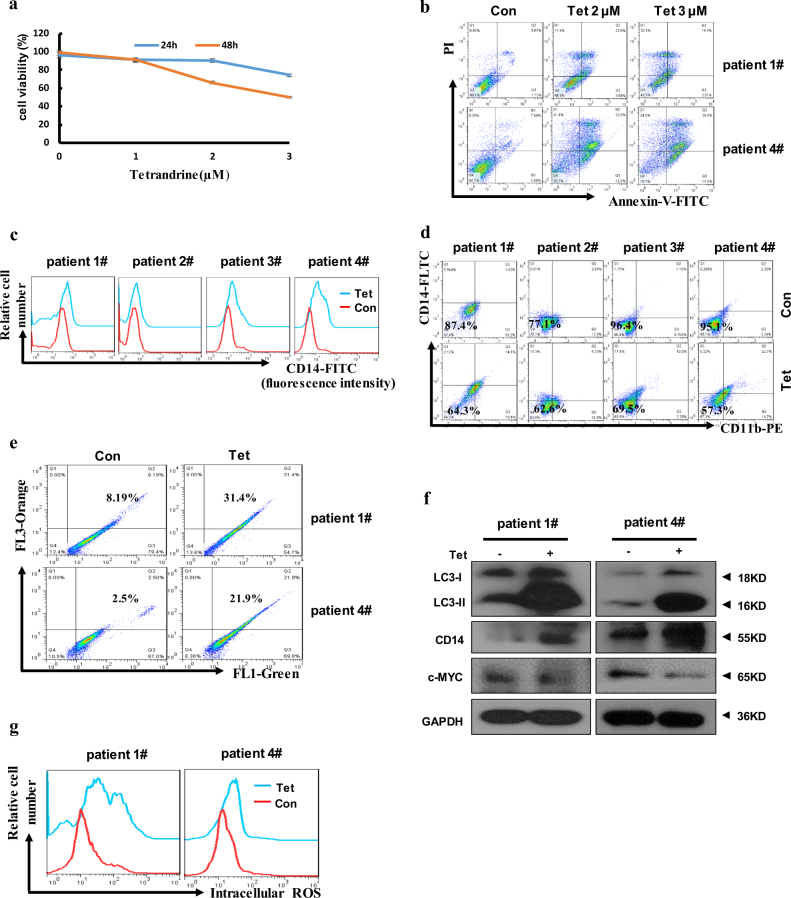
Tetrandrine induced differentiation and autophagy in AML patient cells. (**a**) Cell viability was analyzed by trypan blue dye-exclusion assays. The bars indicate the S.D. *n* = 3. (**b**) Patient #1 and #4 apoptotic cells were analyzed by flow cytometry after treatment with tetrandrine at the indicated concentrations. (**c**) Analysis of CD14 or (**d**) CD14 and CD11b expression by flow cytometry after DMSO or 2 μM tetrandrine treatment in all patient cells for 3 days. (**e**) The patient #1 and #4 cells were treated with DMSO or 2 μM tetrandrine for 24 h, stained with acridine orange, and then analyzed by flow cytometry. (**f**) Western blot analysis of LC3, CD14, c-MYC and GAPDH in tetrandrine-treated #1 and #4 patient cells. (**g**) After 24 h treatment, the intracellular ROS levels were assessed via flow cytometry analysis in #1 and #4 patient cells

To determine the effects of tetrandrine on human primary leukemia cells, here, cells were obtained from four AML patients who received no chemotherapy. Because of the small number of cells isolated from the patient 2# and 3# blood samples, apoptosis, acridine orange staining, western blot and ROS levels analysis were just validated in patient 1# and 4#. As shown in Figs. S6A, 2–3 μM tetrandrine can inhibit cell proliferation. When the cells were treated with the indicated concentrations of tetrandrine, trypan blue dye-exclusion assay and Annexin-V/PI staining showed that tetrandrine had significant anti-leukemia effects (Fig. 7a, b). Next, we found tetrandrine facilitated increase CD14 and CD11b expression on the surface of patient cells compared to vehicle treatment (Fig. 7c, d). As shown in Fig. 7f, western blot results also showed that tetrandrine treatment promoted CD14 expression and inhibited c-MYC levels. In addition, tetrandrine-treated cells dramatically increased the percentage of acridine orange-positive cells and up-regulated LC3-II levels (Fig. 7e, f). And we also found that the intracellular ROS levels were significantly increased in the tetrandrine-treated patient cells (Fig. 7g). Tetrandrine-induced CD14 expression was also prevented by NAC and 3-MA in the patient cells (Figure S6B and C). These results revealed that tetrandrine may effectively regulated proliferation, apoptosis, differentiation, autophagy and ROS accumulation in primary leukemia cells. Moreover, ROS activation and autophagy may be associated with tetrandrine-induced differentiation.

### Tetrandrine at 2 μM had no cytotoxicity or effects on differentiation in human CD34+ progenitor cells

**Fig. 8:**
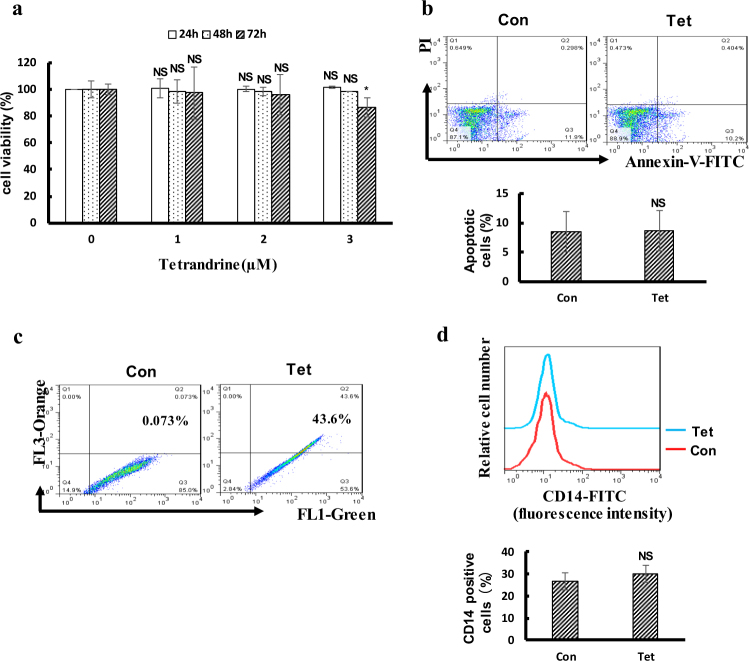
Tetrandrine at 2 μM had no toxicity on human CD34 + progenitor cells. The bars indicate the S.D. *p < 0.05. (**a**) Cell viability of human CD34+ progenitor cells was assessed by MTS assay. (**b**) Apoptotic cells were detected by flow cytometry in CD34+ cells following treatment with DMSO or 2 μM tetrandrine for 48 h. (**c**) After 24 h of treatment with DMSO or 2 μM tetrandrine, cells were stained with acridine orange and analyzed by flow cytometry. (**d**) The expression of CD14 in the CD34 + cells was measured by flow cytometry after treated with DMSO or 2 μM tetrandrine for 4 days. NS not significant

In this study, the effect of 2 μM tetrandrine on human CD34+ progenitor cells has been studied. As shown in Fig. 8, the results showed that 2 μΜ terandrine has no effect on human CD34+ progenitor cells viability and differentiation but may be trigger autophagy. In addition, we also found that tetrandrine has no significant effects on cell viability and CD14 expression but triggered autophagy of healthy mouse haematopoietic stem cells (Figure S7).

## Discussion

Studies have demonstrated that induced cell differentiation can effectively treat leukemia^30–34^. However, the side effects of drugs are of primary consideration. Based on a long history of clinical applications of traditional Chinese medicine, tetrandrine is considered to be a safe agent. Tetrandrine has been used for cancer chemoprevention and therapy^14,35,36^. In this study, we demonstrated that tetrandrine can inhibit leukemia cell proliferation and induce differentiation. Mechanistically, tetrandrine-induced differentiation was mainly associated with the stimulation of ROS and autophagy. Moreover, inhibited c-MYC protein expression played a critical role in tetrandrine-induced leukemia cell differentiation.

Most normal cell growth and development requires a well-controlled balance of protein synthesis and organelle biogenesis to protein degradation and organelle renewal. The major pathways for the degradation of cellular constituents are autophagy and cytosolic turnover by the proteasome^37^. Differentiation of cells is usually associated with slowed cell growth, which is caused by an altered rate of macromolecule synthesis and degradation. Research has shown that autophagy can regulate myeloid cell differentiation^22,38^. In this study, we observed that early autophagy mediated the tetrandrine-induced differentiation. Further inhibition of autophagy by knockout of ATG7 gene decreased tetrandrine-induced differentiation in K562 cells. Moreover, others have reported that autophagy is required for differentiation of nonhematologic cells and tissues. Together, these results show that autophagy is important for cell differentiation.

ROS have emerged in recent years as important regulators of cell division and differentiation^39^. There are reports that ROS are produced in the early stage of monocyte–macrophage differentiation. ROS generation blockade induced by BHA, TEMPO, NAC and apocynin specifically inhibits M2 macrophage differentiation^40,41^. In the present study, we observed that tetrandrine treatment significantly stimulated ROS generation. We also observed that the ROS scavengers NAC and Tiron can inhibit tetrandrine-induced leukemia cell autophagy and differentiation. These results suggest that tetrandrine accelerated intracellular ROS generation that is important for tetrandrine-induced autophagy and differentiation.

The MYC oncogene contributes to the genesis of many human cancers^42^. Many reports have indicated that c-MYC protein can regulate terminal differentiation of hematopoietic cells^43–45^. In our study, tetrandrine was shown to reduce c-MYC protein expression. Then, overexpression of c-MYC in K562 and THP-1 cells inhibited tetrandrine-induced cell differentiation, and animal experiments also validated these results. Moreover, we found that 40 μM 10058-F4 can enhance tetrandrine-induced differentiation (Fig. 5h) but 80 μM 10058-F4 alone induce K562 cells differentiation (date not shown). However, the signaling pathways involved in tetrandrine-induced differentiation that are modulated by c-MYC remain unclear and will require further investigation.

In summary, we demonstrated that tetrandrine has the potential to treat leukemia. Above all, we discovered that tetrandrine can induce autophagy and differentiation both in vitro and in vivo. The potential molecular mechanisms involve activation of ROS accumulation and inhibition of c-MYC expression. These studies provide the rationale for application of tetrandrine in clinical therapies and in therapeutic regimens for leukemia patients.

## Materials and methods

### Cell lines and cell culture

HL60 cells were purchased from CCTCC (China Center for Type Culture Collection; Wuhan, China). K562, U937 and THP-1 cells were kindly provided by Dr. Zan Huang (Wuhan University). All cell lines were grown in RPMI 1640 medium supplemented with 10% FBS (fetal bovine serum, Hyclone), 1% streptomycin and 1% penicillin. AML patient cells were provided by Dengju Li (Tongji Hospital of Tongji Medical College; Wuhan, China). The informed consent was obtained from all of the examined subjects, and the related studies were approved by the ethics committees of the participating hospitals and institute. AML patient cells were cultured in expansion media (RPMI 1640 with 10% FBS, 10 ng/ml recombinant human IL-3, 10 ng/ml rhIL-6, and 50 ng/ml stem cell factor). All cells were cultured in a humidified atmosphere containing 5% CO_2_ at 37 °C. Cell culture dishes and plates were purchased from Wuxi NEST Biotechnology Co. Ltd. (Wuxi, China).

### Chemical reagents and antibodies

Tetrandrine was acquired from Shanghai Ronghe Medical (Shanghai, China). Wright-Giemsa stain was obtained from Baso (Zhuhai, China). DCFH-DA was from Invitrogen (Carlsbad, CA). Acridine orange, 3-MA, DMSO, NAC, and Tiron were purchased from Sigma-Aldrich (St. Louis, MO). NBT was from Beyotime (Nantong, China). The FITC Annexin V Apoptosis Detection Kit I, CD14-FITC and CD11b-PE were obtained from BD Biosciences. Red blood cell lysis buffer, IL-3, IL6 and stem cell factor were kindly provided by Dr. Zan Huang (Wuhan University). MTS was acquired from Promega (Madison, USA). The antibody against LC3 was from Sigma-Aldrich (St. Louis, MO). The caspase-3, PARP, and ATG7 antibodies were obtained from Cell Signaling Technology (Beverly, MA). Antibodies against CD14 and c-MYC were purchased from Proteintech Group Inc. (Chicago, IL). The anti-GAPDH antibody and the horseradish peroxidase (HRP)-conjugated secondary antibodies (goat–anti-rabbit and goat–anti-mouse) were acquired from Beyotime (Nantong, China).

### Cell proliferation and viability assays

Tetrandrine was dissolved in DMSO to a final concentration of 10 mM and then stored at −80 °C. For proliferation and viability assays, 3000 cells (K562 and HL60) or 5000 cells (THP-1 and U937) per well were seeded in a 96-well plate with 100 μl medium, cultured for 24, 48 and 72 h in the presence of varying concentrations of tetrandrine or DMSO (con). The total cell count was then detected by a hemocytometer, and cell viability was measured as the percentage of living cells demonstrated by the trypan blue dye-exclusion assay according to established protocols.

### Cell cycle analysis

Cells were cultured for 48 h in the presence of 2 μM tetrandrine or DMSO (con). The cells were collected and washed with PBS and then fixed with 70% ethanol for at least 4 h at 4 °C. The fixed cells were collected and washed twice with PBS, suspended in cold PBS containing 50 μg/ml of PI and 100 μg/ml of Rnase A and then kept in the dark for 30 min. The samples were transferred to the flow cytometer (Beckman, Indianapolis, CA, USA) and cell fluorescence were measured. The data were analyzed using FlowJo software (TreeStar, San Carlos, CA, USA).

### Cell apoptosis analysis by flow cytometry

Cells were treated with 2 μM tetrandrine or DMSO (con) for 48 h, collected, washed twice with PBS, resuspended in binding buffer and then dyed with Annexin V-FITC and PI for 15 min in the dark according to the manufacturer’s instructions. Annexin V fluorescence was measured by flow cytometry, and the membrane integrity of the cells was simultaneously assessed by the PI exclusion method.

### Morphological observation

Cell morphology was determined by a Wright-Giemsa staining assay following 4 days of 2 μM tetrandrine treatment. Cells were collected and smeared on microscope slides from Citoglas (Jiangsu, China). After Wright-Giemsa staining, the slides were cleaned gently, observed under a light microscope (CKX41, Olympus Optical Co., Ltd.) and photographed.

### NBT reduction assay

For the NBT reduction analysis, cells were treated with 2 μM tetrandrine for 0, 4, 5 and 6 days. Then, the cell suspensions were incubated with 1 mg/ml of NBT and 20 ng/ml of TPA in an equal volume of RPMI1640 for 30 min at 37 °C. After 30 min, the cells were washed with PBS and resuspended in 100 μl of PBS. NBT was evaluated both as the percentage of positive cells and the intensity of reduction when measured at a wavelength of 570 nm with microplate readers (SpectraMax M5).

### Detection of CD14 and CD11b expression by flow cytometry

Cells were plated in 12-well plates and mixed with 2 μM tetrandrine for indicated time. To measure the expression of differentiation markers CD14 or CD11b, cells were washed twice in cold PBS after dilution to a density of 1 × 10^5^ cells per well, and then incubated with the CD14-FITC and/or CD11b-PE antibody for 20 min in the dark. Samples were washed with cold PBS and measured by flow cytometry.

### Acridine orange staining assay

Acridine orange staining can detect intracellular acidic autophagic vacuoles by flow cytometry or fluorescence microscopy. For the acridine orange staining assay, the cells were plated in 12-well plates and treated with 2 μM tetrandrine for 24 h. Cells were then stained with acridine orange (1 μg/ml) at 37 °C for 25 min before observation. The red acidic vesicular organelles (AVOs) in autophagy cells were measured by flow cytometry or visualized by fluorescence microscopy.

### Measurement of intracellular ROS levels

The cell-permeant probe, DCFH-DA, which fluoresces when it is oxidized, was used to measure intracellular ROS levels. Cells were treated with DMSO or the indicated concentrations of tetrandrine in a 12-well plate for 12 h and 24 h. Then, cells were collected and washed with PBS and resuspended in 500 μl of serum-free RPMI 1640 medium containing 0.5 μl DCFH-DA at 37 °C for 30 min. The prepared cells were evaluated using flow cytometry.

### Western blot analysis

After treatment, cells were collected and washed with PBS and then lysed in 1% sodium dodecyl sulfate (SDS) on ice. Cell lysates were heated to 98 °C for 15 min and then centrifuged at 12,000×*g* for 15 min. The supernatant was collected, and protein concentrations were assessed using the Bicinchoninic Acid Protein Assay Kit (Thermo scientific). Equal amounts of protein were separated by SDS–PAGE and transferred to a PVDF membrane (Millipore), which was then immunoblotted with the indicated antibodies.

### Quantitative real-time PCR

Cells were treated with 2 μM tetrandrine or DMSO for 24 h. Total RNA was isolated using the Total RNA Kit I (Omega Bio-Tek, Inc., GA). Then, RNA was transcribed into cDNA using the Transcriptor First Strand cDNA Synthesis Kit (Roche Life Science, USA) according to the manufacturer’s instructions. qRT–PCR was performed using the FastStart Universal SYBR Green Master kit (Rox) (Roche Life Science, USA) on the Applied Biosystems 7500 Fast Real-Time PCR System (PerkinElmer, Torrance, CA). The following primer pairs were used for qRT–PCR: c-MYC: forward, 5′-CACCGAGTCGTAGTCGAGGT-3′ and reverse, 5′-TTTCGGGTAGTGGAAAACCA-3′. GAPDH: forward, 5′-TCCACCACCCTGTTGCTGTA-3′ and reverse 5′-ACCACAGTCCATGCCATCAC-3′. All reactions were performed in triplicate in a 20-μl reaction volume. Fold changes in gene expression were determined using the 2^−^^ΔΔCt^ method with GAPDH as an endogenous control.

### Plasmids and transient transfection

The GFP-LC3 plasmid was kindly provided by Dr. Tamotsu Yoshimori (National Institute of Genetics, Mishima, Japan). The pEGFP-mRFP tandem fluorescent-tagged LC3 (ptfLC3) plasmid was purchased from Addgene (Cambridge, MA). To detect autophagy and autophagic flux, the cells were seeded in a 12-well plate, then transfected with the GFP-LC3 or ptfLC3 plasmids for 24 h, and then treatment with indicated drugs to present autophagy and autophagic flux. After treatment the cells were collected and observed with a fluorescence microscope (Olympus BX51).

### Plasmids and lentiviral transfection

The pHAGE.puro-c-MYC plasmid and the empty vector plasmid were kindly provided by Dr. Li Y (College of Life Sciences, Wuhan University, China). Transfection reagents were kindly provided by Dr. Xiaodong Zhang (College of Life Sciences, Wuhan University, China). In our study, HEK-293T cells were transfected with either the c-MYC plasmid or an empty vector together with the pMD2.G and psPAX2 plasmids using transfection reagents. At 48 h post-transfection, the supernatants were collected and filtered with 0.45-μm filters. Then, K562 and THP-1 cells were cultured with the supernatants containing 5 μg/ml polybrene. After 24 h, the virus-containing medium was replaced by fresh medium with 2 μg/ml puromycin. Stable cells were selected with puromycin.

### Tumor xenograft model

The xenograft model in athymic nude mice was performed to evaluate the in vivo efficacy of tetrandrine. Animal experimental protocols and care were approved by the Experimental Animal Center of Wuhan University. Male nude mice of 4 to 5 weeks of age were obtained from the Model Animal Research Center (Changsha, China). THP-1 cells (~1 × 10^7^ cells) and the same number of THP-1 cells overexpressing c-MYC in a total volume of 0.2 mL of PBS were inoculated subcutaneously over the right flank of each mouse. Tumor diameter and body weights were measured every day. Tumor volume was calculated by the following formula: 0.52 × length × width^2^. Mice were randomized into three groups (five per group) when the tumor volume reached ~50 mm^3^. There were two treatment groups of mice that received tetrandrine at 25 or 50 mg/kg, while the other mouse group was given a vehicle treatment of 0.5% methylcellulose.

### Malondialdehyde (MDA) assay

Mice were killed after 13 days of tetrandrine treatment, and tumor tissues were removed. For the MDA assay, tissue proteins were prepared according to the description in the Lipid Peroxidation MDA assay kit (Beyotime, Nantong, China). The MDA levels were evaluated by Multi-Mode Microplate Readers (SpectraMax M5) at 532 nm using 490 nm as a control.

### Immunohistochemistry

The tumor tissue sections were fixed by 4% paraformaldehyde, embedded in paraffin and sliced at a 5 μm thickness for immunohistochemical analysis. After deparaffinization and the appropriate epitope retrieval, the sections were stained with c-MYC, CD14 and LC3 antibodies and further incubated with biotinylated goat–anti-rabbit antibodies. The specific signals were then detected with streptavidin-conjugated HRP and diaminobenzidine as the chromogen.

### Healthy progenitor cell study

Mouse hematopoietic stem cells and human CD34+ progenitor cells were used to measure tetrandrine drug toxicity. ICR mice were obtained from the Disease Prevention Center of Hubei Province. In the experiment, mice embryos were collected after 12.5 days of conception and the fetal livers were titrated with a pipette needle to obtain a single-cell suspension. Human CD34+ progenitor cells were provided by Dr. Zan Huang (Wuhan University). Then, cells were cultured with complete RPMI 1640 media containing IL-3 and IL6 at 10 ng/ml and stem cell factor at 100 ng/ml. To induce the cells to differentiate, cells were cultured in differentiation media (RPMI 1640 with 10% FBS, 10 ng/ml stem cell factor and 50 ng/ml GM-CSF). In this study, mouse cells used mouse cytokines and human cells with human cytokines.

### Statistical analysis

Data are expressed as the mean ± S.D. A two-tailed unpaired Student’s *t*-test was used to analyze data containing two groups unless otherwise specified. Statistical significance was denoted as follows: NS, not significant; *P < 0.05, **P < 0.01 and ***P < 0.001 were deemed statistically significant.

## Electronic supplementary material

Supplementary Figures and Supplementary Figure legends
