# Supplementary material for: c-MYC and reactive oxygen species play roles in tetrandrine-induced leukemia differentiation
Source: Cell Death Dis. 2018 Apr 27;9(5):473. doi: 10.1038/s41419-018-0498-9 (PMC5920096; doi:10.1038/s41419-018-0498-9)
Supplement: Supplementary file 1 — Supplementary Figures and Supplementary Figure legends [file 41419_2018_498_MOESM1_ESM.pdf]

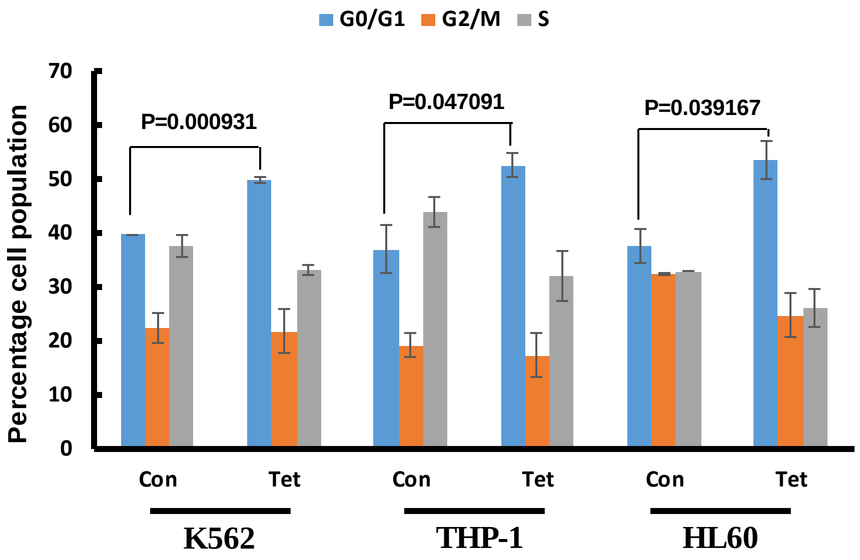

Figure S1. Cell cycle was analyzed by flow cytometry after DMSO or 2μM tetrandrine treatment for 48h, the statistic analysis for three experiments. The bars indicate the S.D.

Figure S1

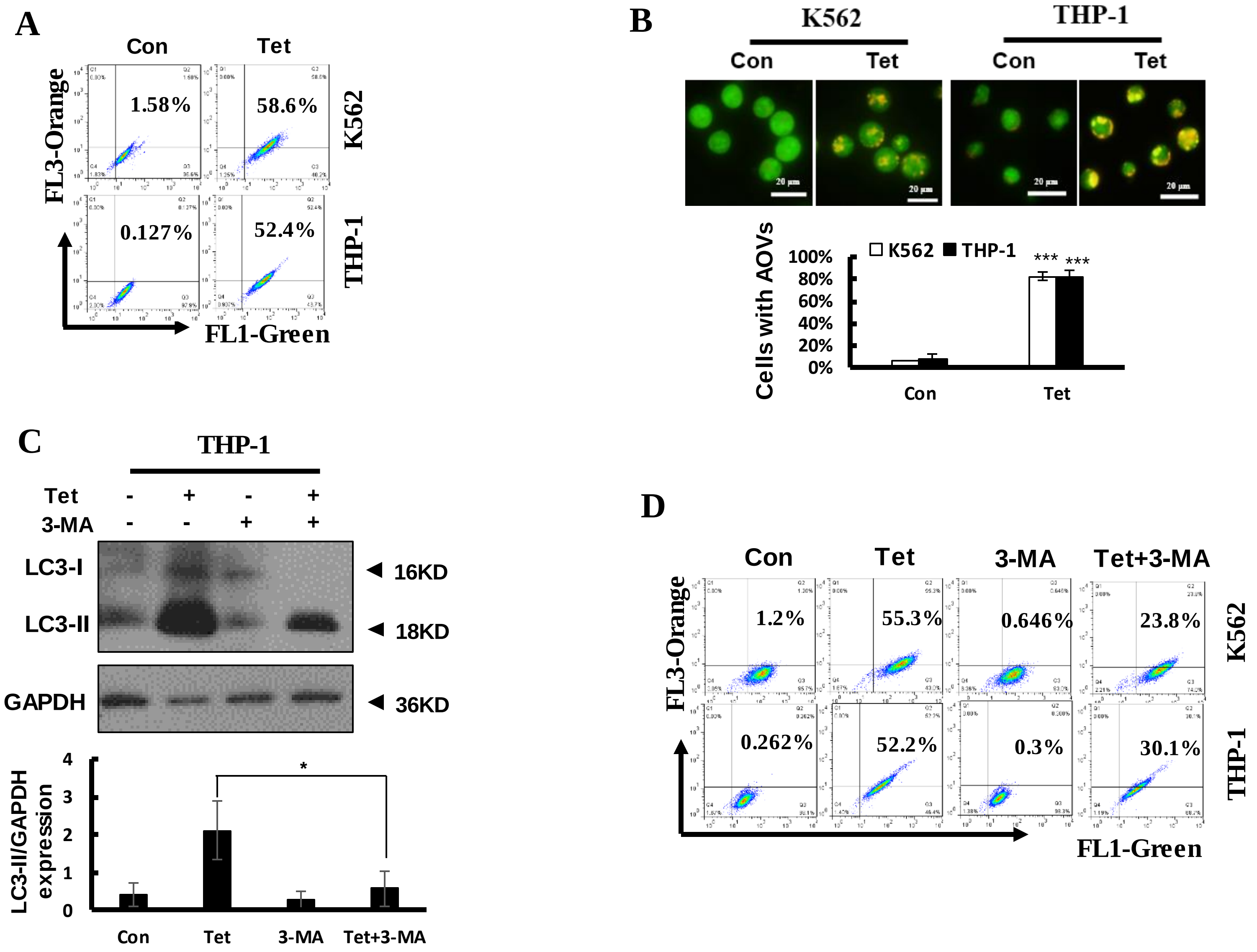
A
Con
Tet
FL3-Orange
1.58%
58.6%
K562
0.127%
52.4%
THP-1
FL1-Green
B
K562
THP-1
Con
Tet
Con
Tet
Cells with AOVs
K562
THP-1
100%
80%
60%
40%
20%
0%
***
***
Con
Tet
C
THP-1
Tet
3-MA
LC3-I
LC3-II
GAPDH
16KD
18KD
36KD
LC3-II/GAPDH expression
4
3
2
1
0
*
Con
Tet
3-MA
Tet+3-MA
D
Con
Tet
3-MA
Tet+3-MA
FL3-Orange
1.2%
55.3%
0.646%
23.8%
K562
0.262%
52.2%
0.3%
30.1%
THP-1
FL1-Green

Figure S2

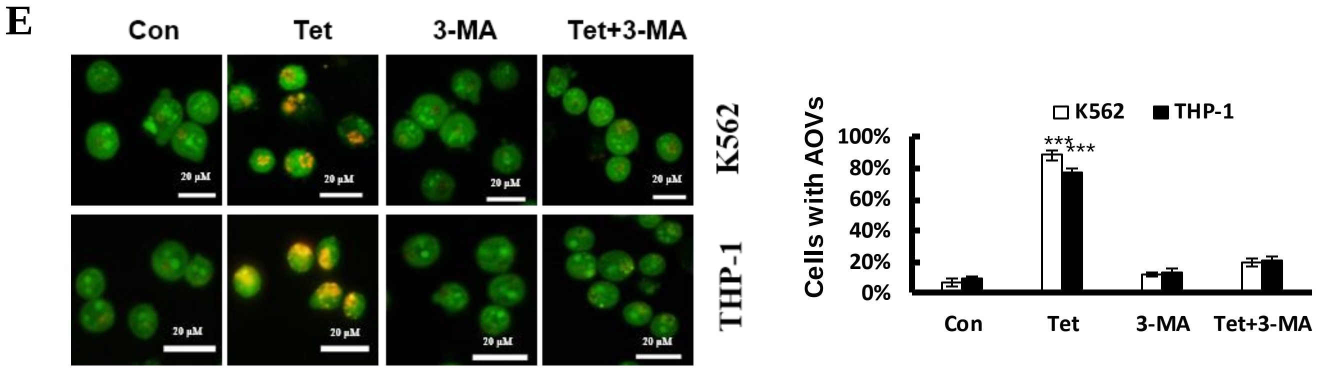

E
Con
Tet
3-MA
Tet+3-MA
K562
THP-1
20 μM
Cells with AOVs
K562
THP-1
100%
80%
60%
40%
20%
0%
***
***
Con
Tet
3-MA
Tet+3-MA

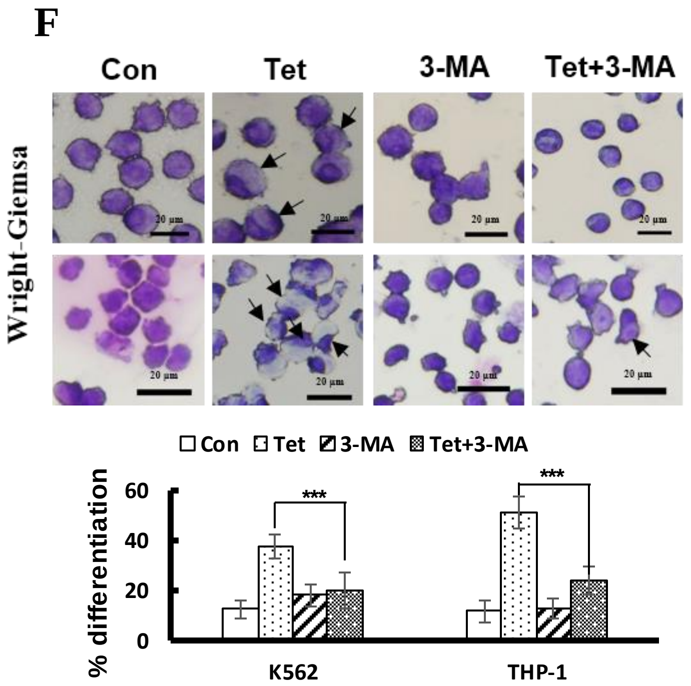

F
Con
Tet
3-MA
Tet+3-MA
Wright-Giemsa
K562
THP-1
20 μm
Con
Tet
3-MA
Tet+3-MA
% differentiation
60
40
20
0
***
***
K562
THP-1

Figure S2

Figure S2. The bars indicate the S.D. *p <0.05, ***p <0.001. A, B, After 24h of DMSO or 2μM Tetrandrine treatment the cells were stained with acridine orange, then analyzed by flow cytometry or visualized by fluorescence microscopy. C. THP-1 cells were treated for 9 h with or without pretreatment 1.5 mM 3-MA for 1 h. Western blot analysis of LC3 levels. D, E. Cells were treated with DMSO or 2μM Tetrandrine for 24h with or without pretreatment with 1.5mM 3-MA for 1h, the light or orange autophagosomes were analyzed by flow cytometry or visualized by fluorescence microscopy. F. Wright-Giemsa staining was used to assess cell morphology after 4 days of treatment with or without pretreatment 1.5 mM 3-MA for 1 h. The arrows indicate lower nucleus/cytoplasm ratio and the cells with horseshoe shape nuclei.

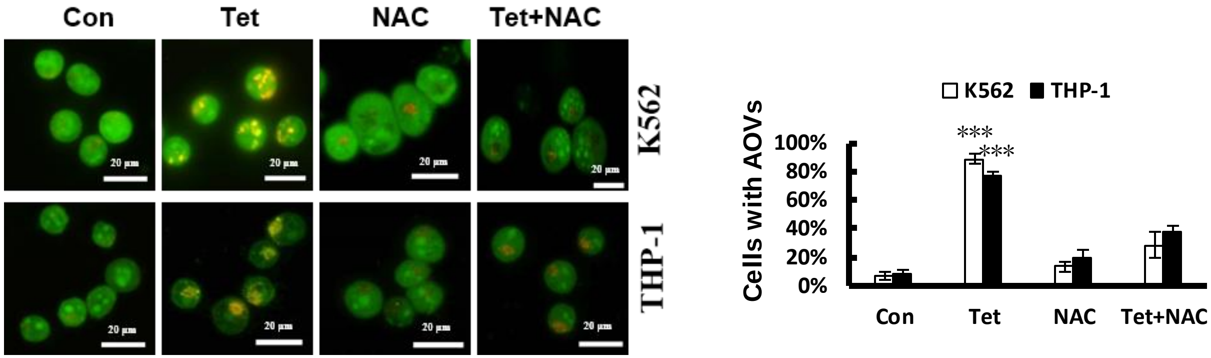

Figure S3. The bars indicate the S.D. ***p <0.001. Cells were treated with DMSO or 2μM Tetrandrine for 24h with or without pretreatment with NAC for 1h, stained with acridine orange then visualized by fluorescence microscopy.

Figure S3

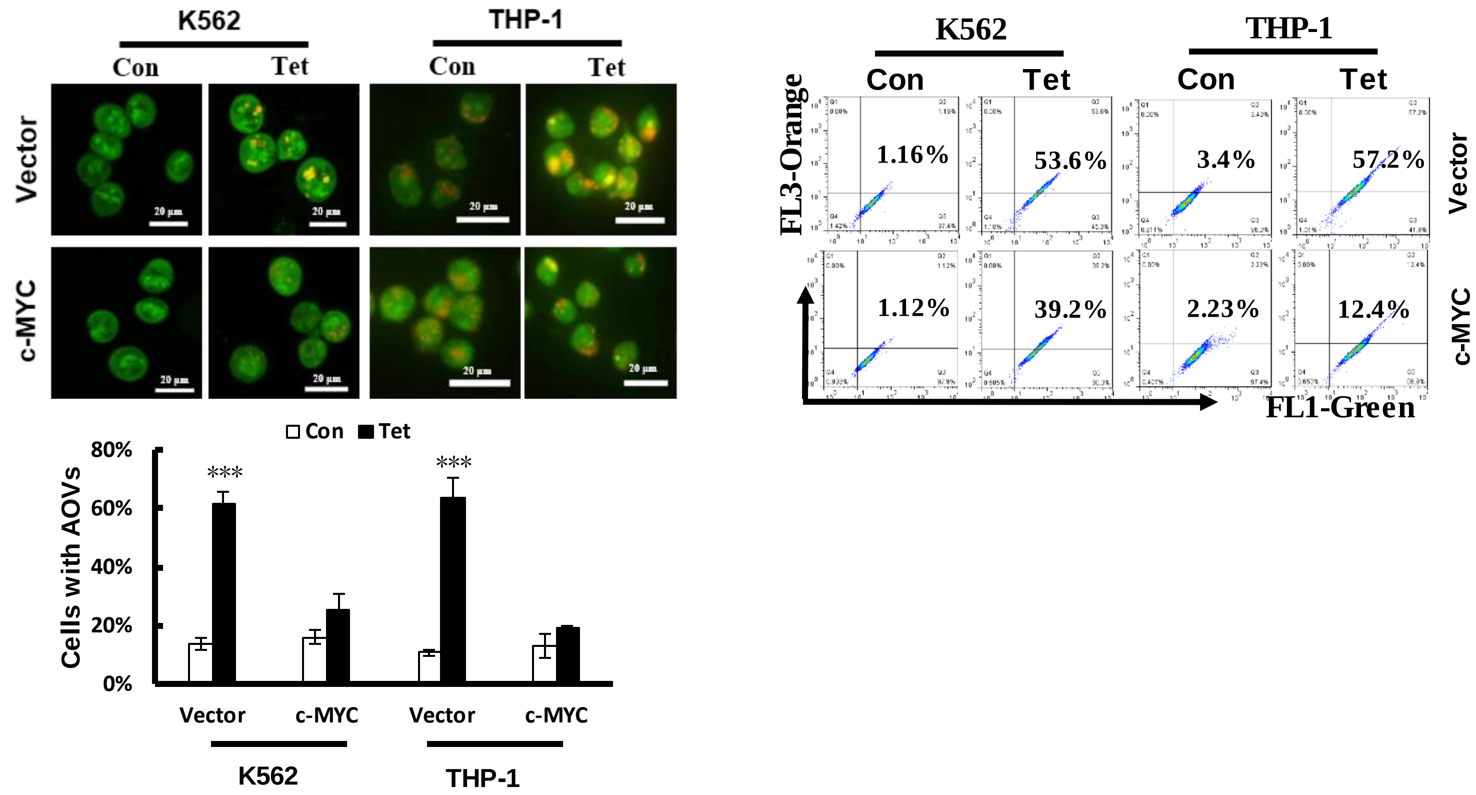

Figure S4. The bars indicate the S.D. ***p <0.001. THP-1 vector or c-MYC cells were treated with DMSO or 2μM Tetrandrine for 24h. The light or orange autophagosomes were observed by fluorescence microscopy or by flow cytometry.

Figure S4

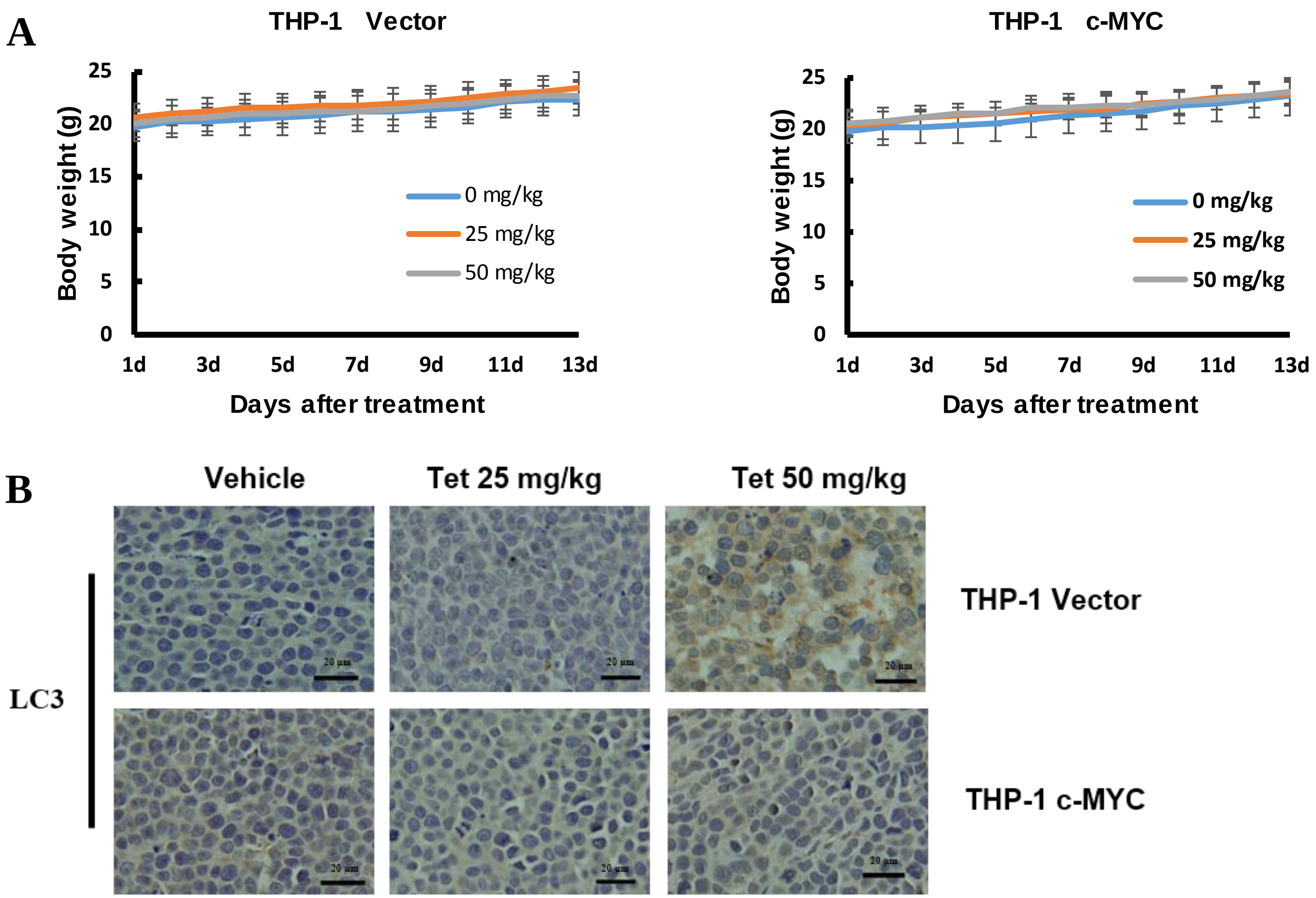

Figure S5. A. Body weights of nude mice bearing established THP-1 vector and THP-1 c-MYC tumor xenografts. B. LC3 was evaluated by immunohistochemistry analysis in tumor tissues. Magnification: × 400.

Figure S5

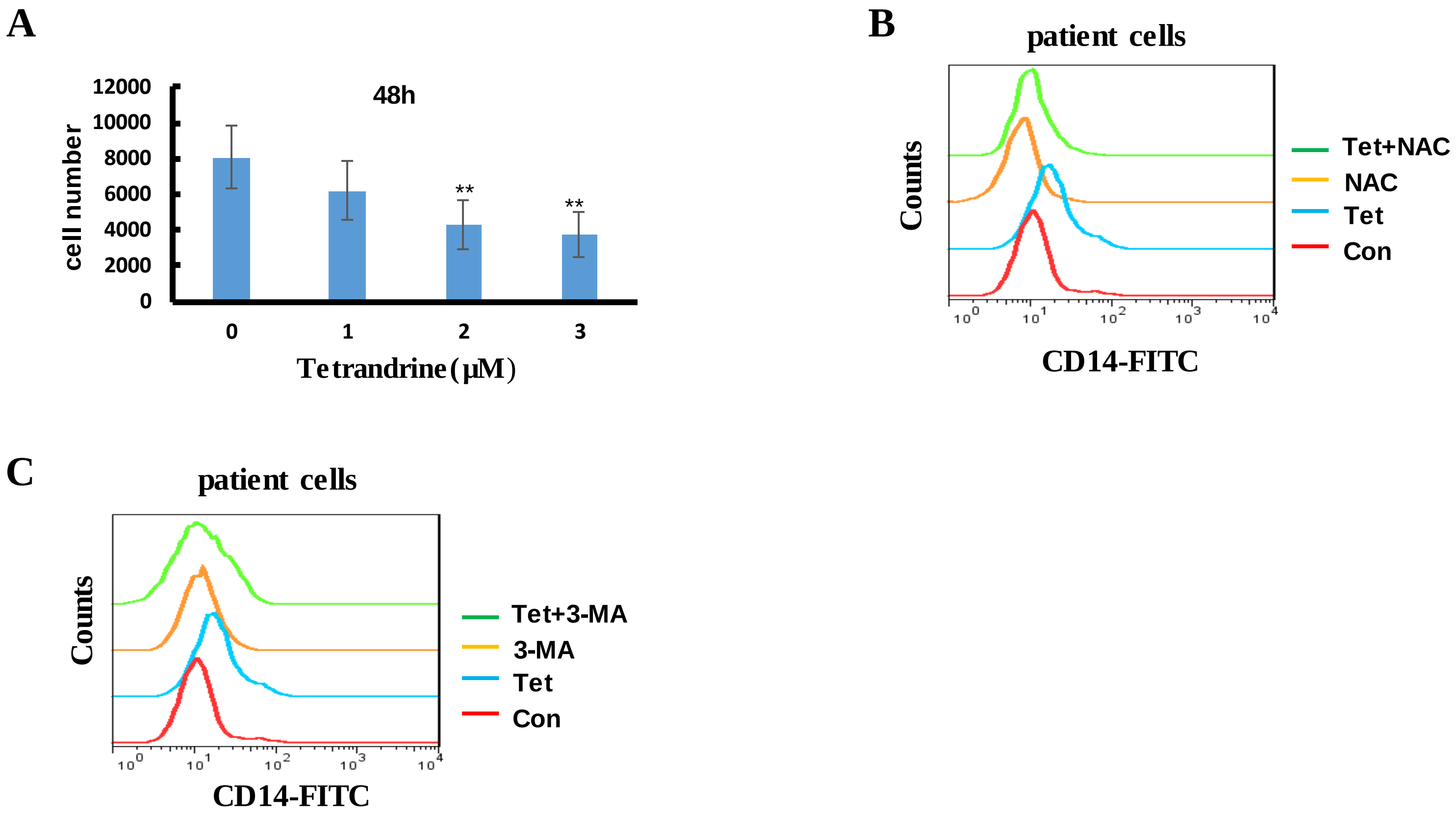

Figure S6. A. Patient' s cells were treated with tetrandrine (0, 1, 2, or 3 μM) for 48 h and then cell proliferation was assessed using a cell counting method. **p<0.01. B,C. After 3 days of DMSO or 2 μM tetrandrine treatment, flow cytometry analyzed CD14 expression with or without pretreatment with 15mM NAC or 1.5 mM 3-MA.

Figure S6

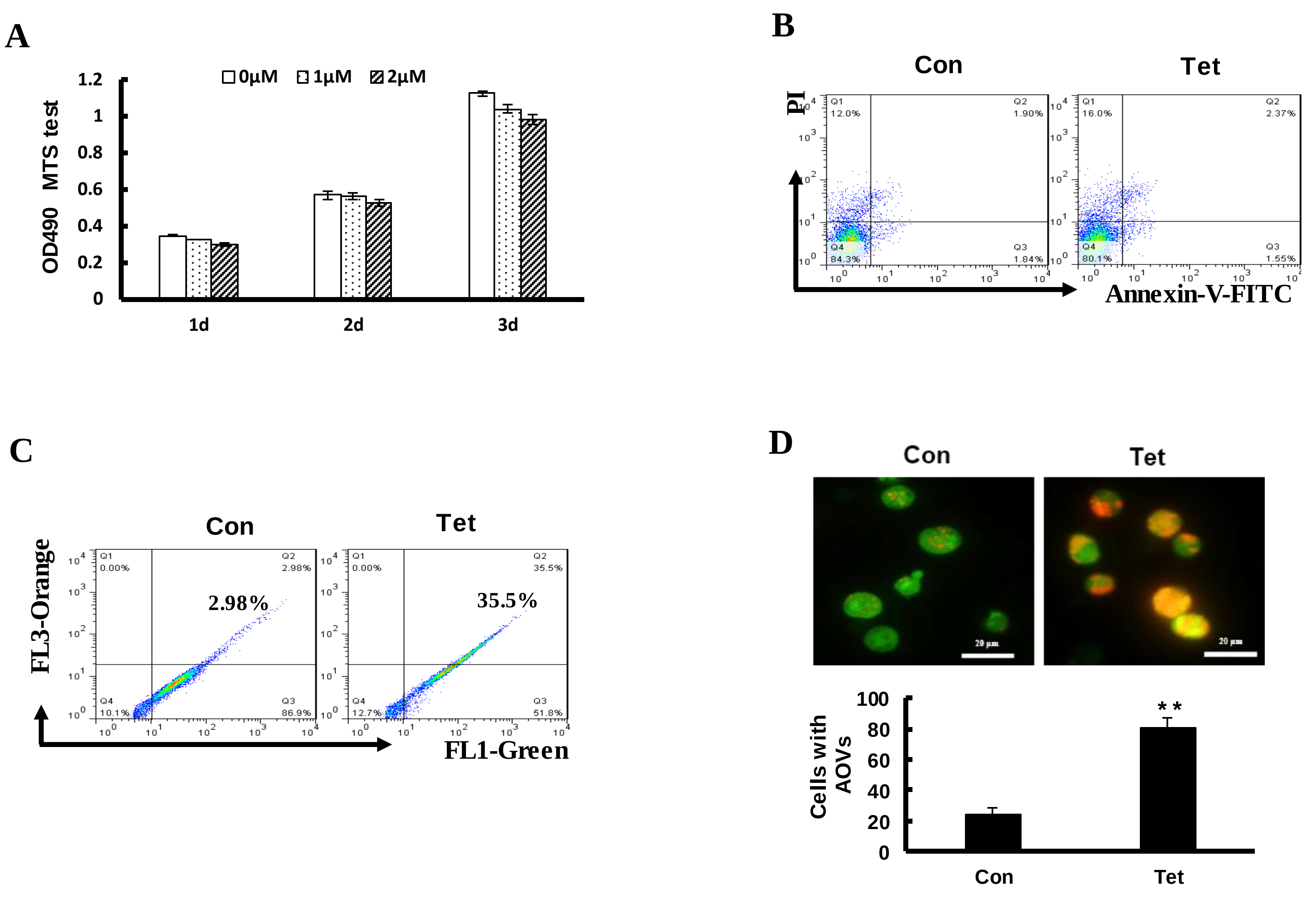
A
0μM 1μM 2μM
OD490 MTS test
1.2
1
0.8
0.6
0.4
0.2
0
1d
2d
3d
B
Con
Tet
PI
Q1 12.0%
Q2 1.90%
Q3 1.84%
Q4 84.3%
Q1 16.0%
Q2 2.37%
Q3 1.55%
Q4 80.1%
Annexin-V-FITC
C
Con
Tet
FL3-Orange
Q1 0.00%
Q2 2.98%
2.98%
Q3 86.9%
Q4 10.1%
Q1 0.00%
Q2 35.5%
35.5%
Q3 51.8%
Q4 12.7%
FL1-Green
D
Con
Tet
20 μm
20 μm
Cells with AOVs
100
80
60
40
20
0
**
Con
Tet

Figure S7

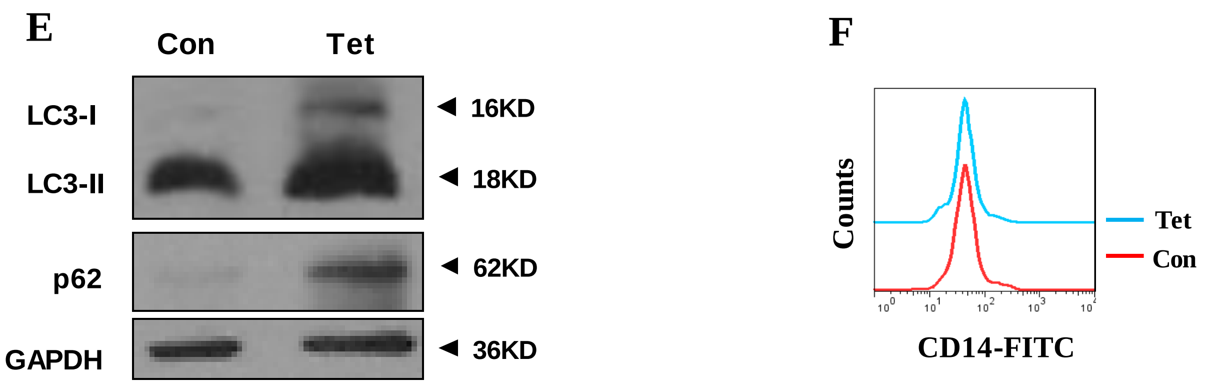

Figure S7. The bars indicate the S.D. A. MTS analysis was performed to determine cell viability. B. Apoptotic cells were detected by flow cytometry in DMSO or 2 μM tetrandrine-treated mouse HSCs. C,D. After 24 h of DMSO or 2 μM tetrandrine treatment, cells were stained with acridine orange and analyzed by flow cytometry or visualized by fluorescence microscopy. n=3. **p<0.01. E. Western blot analysis is shown for LC3, p62 and GAPDH. F. The expression of CD14 in mouse HSCs was measured by flow cytometry after treated with DMSO or 2 μM tetrandrine for 4 days.

Figure S7
